# Single-cell sequencing reveals immune networks and metabolic reprogramming in gynecological malignancies

**DOI:** 10.3389/fimmu.2026.1968253

**Published:** 2026-10-19

**Authors:** Li Ren, Xuemei Li

**Affiliations:** Department of Gynecology, Ziyang Central Hospital, Ziyang, Sichuan, China

**Keywords:** gynecological malignancies, immune network, immunotherapy, metabolic reprogramming, single-cell sequencing, tumor microenvironment

## Abstract

Gynecological malignancies, encompassing ovarian cancer (OC), endometrial cancer (EC), and cervical cancer (CC), represent a group of aggressive neoplasms that pose substantial threats to women’s health worldwide. The complex tumor microenvironment (TME) plays a pivotal role in disease progression and therapeutic resistance. In recent years, single-cell RNA sequencing (scRNA-seq) has enabled systematic characterization of the heterogeneity, functional states, and metabolic reprogramming features of tumor-infiltrating immune cells at unprecedented cellular resolution. Although remarkable progress has been achieved in this field, critical challenges persist, including insufficient understanding of the dynamic mechanisms governing immune cell subset transitions and the intricate regulatory networks underlying metabolism-immune crosstalk. This review summarizes recent advances in single-cell technologies applied to gynecological malignancies, focusing on immune cell subset diversity, intercellular communication networks, and the interplay between metabolic reprogramming and immunosuppression. Furthermore, we discuss potential therapeutic targets and biomarkers derived from these findings, aiming to provide a novel theoretical framework and translational strategies for precision immunotherapy.

## 1 Introduction

Gynecological malignancies, including endometrial, ovarian, and cervical cancers, remain major threats to women’s health worldwide and exhibit substantial inter- and intratumoral heterogeneity (1). Conventional bulk transcriptomic approaches generate averaged molecular profiles that obscure rare cell populations and dynamic cellular states within the TME (2, 3). This limitation is particularly relevant because tumor, immune, stromal, and endothelial cells form highly interactive ecosystems that influence disease progression and therapeutic response (4). Single-cell RNA sequencing (scRNA-seq) has transformed this field by resolving cellular heterogeneity, rare subpopulations, and lineage-specific transcriptional programs at unprecedented resolution (5). Immune composition and functional states are closely associated with clinical outcomes and responses to immune checkpoint inhibitors (6, 7).

Single-cell studies have further revealed dysfunctional CD8^+^ T and natural killer cells, enrichment of regulatory and exhausted T cells, and extensive macrophage plasticity, providing mechanistic insight into immune escape (8–13). Metabolic reprogramming represents another major determinant of TME organization, influencing immune-cell differentiation and function through altered utilization and release of lactate, lipids, amino acids, and other metabolites (14–16). In endometrial cancer, lipid-dependent OLR1/FOXM1/FGF19 signaling contributes to tumor–stromal crosstalk, while N-glycosylation-related programs further shape tumor heterogeneity and immune regulation (17, 18). Emerging single-cell multi-omics approaches integrating transcriptomic, epigenomic, proteomic, metabolic, and spatial information now enable more comprehensive reconstruction of immune–metabolic networks (19–22). Such integrative strategies have identified clinically relevant cell states, including TRAP1l^low^/CAMSAP3^low^ populations associated with aggressive behavior and poor prognosis (23), thereby providing a framework for biomarker discovery and precision therapeutic development.

## 2 Single-cell immune repertoire and epigenetic sequencing in gynecological malignancies

scRNA-seq enables genome-wide transcriptional profiling at single-cell resolution, overcoming the averaging effect of bulk sequencing and revealing cellular heterogeneity within the TME (24, 25). In gynecological malignancies, it has been widely applied to identify tumor-associated immune subsets, drug-resistant populations, and rare cellular states (26, 27). Nevertheless, conventional scRNA-seq lacks spatial information and provides limited direct assessment of protein states, constraining interpretation of immune-cell localization and functional interactions (28). In addition, the marked heterogeneity of gynecological malignancies complicates cell annotation and requires robust computational approaches (29). Single-cell V(D)J sequencing further characterizes adaptive immunity by resolving T-cell receptor and B-cell receptor clonotypes, thereby enabling assessment of lymphocyte clonal expansion and antigen-specific responses (29). Complementary single-cell ATAC-seq and methylation profiling reveal epigenetic programs governing immune-cell activation, exhaustion, exclusion, and immune evasion (30, 31). Integrating transcriptomic, proteomic, and epigenomic data provides a more comprehensive view of immune–metabolic states and regulatory networks (19, 32), while joint scRNA-seq/scATAC-seq analysis links chromatin accessibility to immune-related gene expression and regulatory elements (19, 33). Computational pipelines incorporating dimensionality reduction, trajectory inference, and CellChat-based communication analysis facilitate reconstruction of immune-cell states and interactions (34). inferCNV assists in distinguishing malignant from non-malignant populations (35, 36), whereas AUCell and ligand–receptor analysis help define metabolism–immunity crosstalk (37, 38). Gene regulatory network analysis can further identify transcription factors driving tumor-associated immune programs and potential therapeutic vulnerabilities (39).

## 3 Single-cell atlas of the immune microenvironment in gynecological malignancies

### 3.1 Heterogeneity of tumor-infiltrating t-cell subsets in gynecological malignancies

Single-cell and spatial profiling demonstrates that tumor-infiltrating T-cell heterogeneity is organized differently across OC, EC, and CC. In high-grade serous OC, CD8^+^ T cells span GZMK^+^ memory-like, cytotoxic, progenitor-exhausted, and terminally exhausted states, but their distribution is strongly site dependent. Ovarian lesions may contain few, predominantly dysfunctional T cells together with FOXP3^+^ regulatory T cells (Tregs), whereas omental lesions can be enriched in non-tumor-specific bystander cells; malignant ascites can serve as a reservoir of memory T cells that seed exhausted CD8^+^ and TH1-like populations in solid lesions (40–42). In EC, T-cell composition is more closely coupled to molecular subtype: POLE-mutated and mismatch-repair-deficient tumors carry high neoantigen loads and greater CD8^+^ infiltration with compensatory PD-1/PD-L1 expression, whereas many p53-abnormal or no-specific-molecular-profile tumors are less inflamed (10, 43, 44). CXCL13^+^ tissue-resident/cytotoxic T cells and T follicular helper-like cells also connect T-cell activity to B-cell recruitment and tertiary lymphoid structures (45). In HPV-driven CC, persistent viral antigen exposure supports expanded cytotoxic clonotypes but simultaneously promotes progressive dysfunction characterized by PDCD1, TIGIT, LAG3, and HAVCR2 expression; Tregs and CXCL13^+^ helper populations become increasingly prominent during invasion and nodal dissemination (20, 46, 47). Thus, OC is distinguished by anatomic compartmentalization and ascites-driven trafficking, EC by genotype-dependent immunogenicity, and CC by the coexistence of virus-directed clonal activation and chronic-antigen exhaustion.

### 3.2 Polarization states of tumor-associated macrophages and myeloid-derived suppressor cells

Tumor-associated macrophages (TAMs) and myeloid-derived suppressor cells (MDSCs) form cancer-type-specific suppressive compartments. OC contains monocyte-derived SPP1^+^ macrophages with angiogenic, matrix-remodeling, and lipid-handling programs, together with C1QC^+^ or tissue-resident populations whose abundance differs among primary tumors, omental metastases, and ascites (41, 48, 49). EC likewise contains tumor-enriched macrophage states associated with invasion and immunotherapy resistance, but their polarization is additionally shaped by obesity-related lipids, estrogen signaling, and tumor-derived lactate (13, 15, 50). In CC, single-cell studies resolve C1QC^+^ phagocytic macrophages, inflammatory IL1B^+^ monocyte-like cells, and MRC1/CD163-high immunoregulatory TAMs; the latter become prominent in metastatic lymph nodes, while SPP1-, VEGFA-, and matrix-remodeling programs support angiogenesis and immune exclusion (20, 46, 51). Cervical tumors also contain monocytic and polymorphonuclear suppressor states that overlap transcriptionally with monocytes and neutrophils. S100A8, ARG1, CXCR2, IL1B, and NOS2-associated programs are consistent with arginine depletion, reactive oxygen/nitrogen production, and impaired T- and NK-cell function. Notably, scRNA-seq alone cannot reliably distinguish bona fide MDSCs from conventional neutrophils or monocytes, these assignments require flow cytometry and functional suppression assays (**Figure 1**).

**Figure 1:**
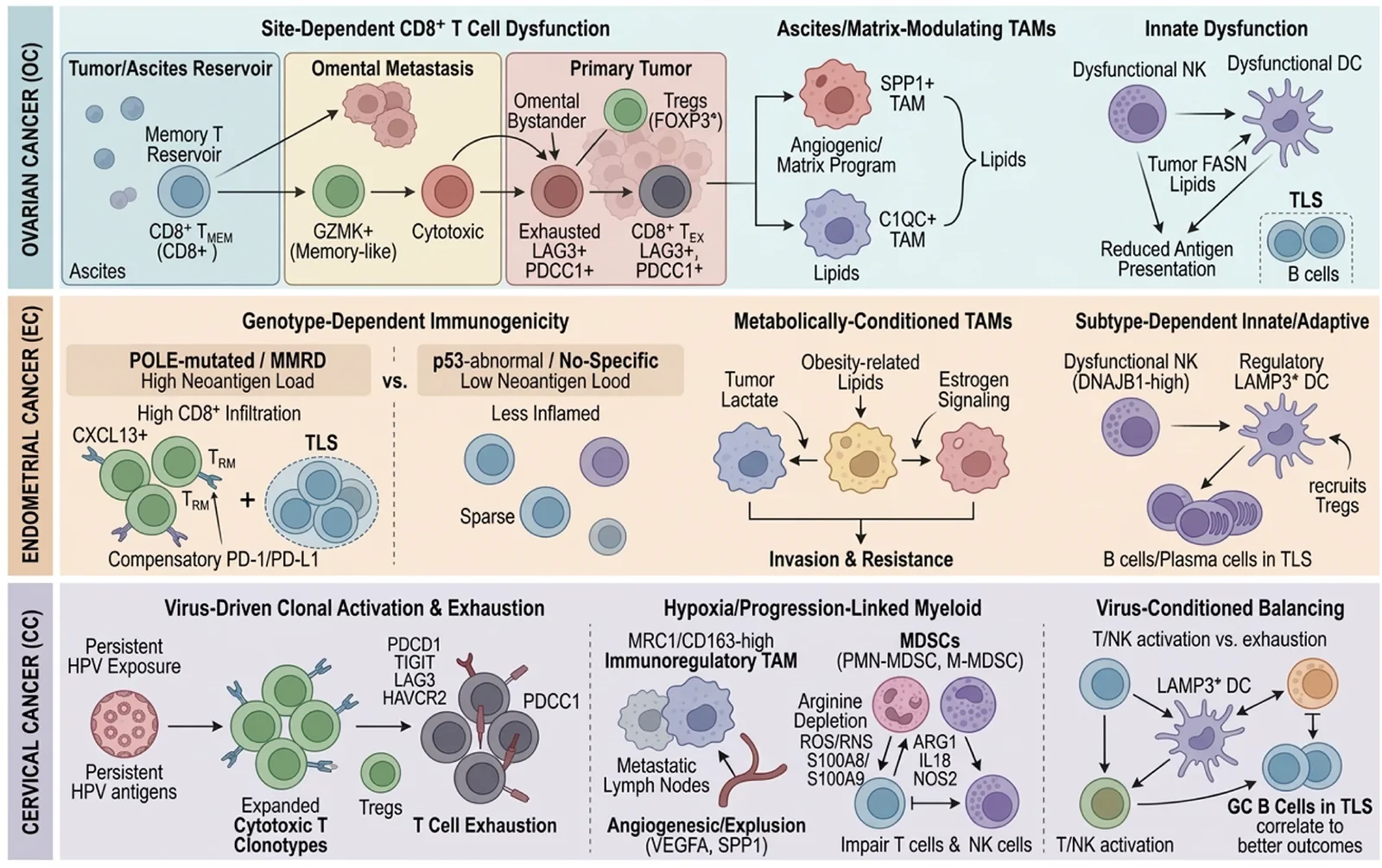
Single-cell atlas and distinct immune microenvironment landscapes in gynecological malignancies.

### 3.3 Immune network participation of natural killer cells, dendritic cells, and B cells

Natural killer (NK) cells, dendritic cells (DCs), and B cells participate in distinct but interconnected immune networks across the three malignancies. In OC, NK cells in ascites and tumors frequently display reduced cytotoxicity, while lipid-rich tumor products and tumor-intrinsic fatty-acid synthase impair DC antigen presentation; conversely, coordinated B-cell, plasma-cell, and T-cell infiltration within TLS is associated with more favorable immune states (41, 52, 53). EC contains a dysfunctional CD56^dim^ DNAJB1^high^ NK subset with stress-response features and strong predicted interactions with TAMs. LAMP3^+^ mature regulatory DCs can recruit Tregs, inhibit CD8^+^ T cells, and provide inadequate activation to NK cells, whereas B-cell differentiation into germinal-center and antibody-secreting states is concentrated in TLS, particularly in immunogenic POLE-mutated and mismatch-repair-deficient tumors (11, 45). CC differs because viral antigens can sustain cytotoxic T/NK activation and germinal-center reactions even as chronic HPV exposure drives exhaustion. Tumor-infiltrating LAMP3^+^ DCs connect T cells and B cells through chemokine and costimulatory networks, and germinal-center B-cell/TLS signatures correlate with improved outcome and pembrolizumab responsiveness (20, 46). Therefore, OC is dominated by ascitic and lipid-mediated innate dysfunction, EC by molecular-subtype-dependent coordination between dysfunctional NK cells, regulatory DCs, and TLS-associated B cells, and CC by a virus-conditioned balance between active antigen presentation and chronic immune exhaustion (**Supplementary Table S1**).

## 4 Intercellular communication networks in the gynecological malignancies microenvironment

### 4.1 Ligand-receptor interaction analysis reveals immunosuppressive signaling networks

Ligand-receptor inference reveals shared suppressive circuits but different dominant cellular senders across OC, EC, and CC. In OC, tumor cells and fibroblasts use CXCL16-CXCR6 and CXCL12/CXCL14-CXCR4 axes to regulate T-cell recruitment or exclusion, whereas SPP1-CD44, MIF-CD74/CD44, TGF-beta-TGFBR, and LGALS9-HAVCR2 signaling connect malignant cells, macrophages, and exhausted lymphocytes (41, 42, 57). In EC, malignant epithelial cells and stromal cells preferentially engage macrophages through CSF1-CSF1R and MIF-CD74/CD44, while CXCL12-CXCR4 and TGF-beta pathways reinforce stromal retention and T-cell dysfunction; LAMP3^+^ DC-derived CCL17/CCL22 signals may additionally recruit CCR4^+^ Tregs (11, 12, 58). In CC, progression from premalignant lesions to invasive and nodal disease is accompanied by intensified MIF-CD74, SPP1-CD44, CXCL12-CXCR4, CCL20-CCR6, and VEGF-receptor communication, together with BAFF/APRIL signals that support B-cell and plasma-cell niches (20, 46, 47, 51). These comparisons indicate that macrophage-centered circuits are common to all three diseases, but OC emphasizes compartment-specific stromal chemokines, EC emphasizes epithelial-myeloid and regulatory-DC communication, and CC integrates HPV-driven inflammation with myeloid, vascular, and lymphoid signaling.

### 4.2 Metabolic-communication interactions between cancer-associated fibroblasts and immune cells

Cancer-associated fibroblasts (CAFs) couple nutrient exchange to immune regulation in each gynecological malignancy, although the relevant tissue context differs. In OC, inflammatory and myofibroblastic CAF states produce CXCL12/CXCL14, IL-6, and TGF-beta, creating T-cell-excluded regions and recruiting suppressive myeloid cells. Glycolytic CAFs also release lactate, while stromal CD39/CD73 generates extracellular adenosine; these metabolic products impair NK- and T-cell function and are reinforced by fatty-acid transfer from omental adipocytes (42, 59–61). In EC, CAF programs operate within an obesity- and hormone-sensitive organ. IL-6/CXCL12 and TGF-beta signaling promotes myeloid recruitment and Treg activity, whereas competition for glucose and the exchange of lactate and lipids can favor malignant epithelial cells over cytotoxic lymphocytes; these effects are expected to be strongest in metabolically dysregulated tumors (17, 50, 62). In CC, inflammatory CAFs and contractile CAFs become spatially associated with hypoxic, glycolytic tumor regions and metastatic lymph nodes. Their CXCL12, TGF-beta, IL-6, and extracellular-matrix programs restrict immune entry, while lactate-rich conditions favor suppressive macrophage and MDSC states (51, 63, 64). Thus, OC CAF biology is shaped by ascites and omental fat, EC CAF biology by endocrine-metabolic exposure, and CC CAF biology by HPV-associated inflammation, hypoxia, and treatment-induced tissue remodeling.

### 4.3 Formation of tertiary lymphoid structures and construction of anti-tumor immune networks

TLS are present in all three malignancies, but their maturation, location, and cellular composition determine whether they support effective antitumor immunity. In OC, CXCL13-producing CD4^+^ T cells initiate early lymphoid aggregates, whereas mature TLS contain organized B-cell follicles, follicular dendritic cells, plasma cells, and cytotoxic T cells. Their activity varies between ovarian and omental sites and is constrained by surrounding stroma; mature B-cell-rich TLS are associated with clinically favorable T-cell phenotypes (52, 54, 55). In EC, single-cell analysis resolves naive, germinal-center/cycling, and antibody-secreting B-cell states within TLS. Mature TLS occur disproportionately in POLE-mutated and mismatch-repair-deficient tumors, and L1CAM expression in germinal centers provides a practical histologic marker associated with favorable prognosis independent of molecular subgroup (45). In CC, HPV-associated chronic inflammation supports CXCL13^+^ T follicular helper cells, germinal-center B cells, plasma cells, and activated antigen-presenting cells. TLS-positive tumors show enhanced macrophage activation and immune-checkpoint expression and generally have improved outcomes, suggesting an inflamed but counter-regulated niche (20, 56). This framework explains why TLS coexists with checkpoint expression and supports combining TLS-based stratification with immune-checkpoint blockade rather than interpreting TLS as uniformly protective.

## 5 Metabolic reprogramming and metabolic-immune crosstalk in the gynecological malignancies microenvironment

### 5.1 Characteristics of metabolic reprogramming in tumor cells

Tumor-cell metabolic reprogramming is shared across gynecological malignancies but reflects different anatomic and etiologic pressures. OC combines aerobic glycolysis with pronounced oxidative and lipid plasticity. HIF-1alpha-driven glucose uptake and lactate production coexist with fatty-acid acquisition from omental adipocytes, *de novo* lipogenesis, and glutamine-dependent anaplerosis, enabling dissemination through nutrient-variable ascites and metastatic sites (65, 66). EC is tightly linked to systemic insulin resistance and excess free fatty acids. PI3K-AKT-mTOR activation, glycolysis, *de novo* lipogenesis, and endoplasmic-reticulum stress cooperate to support growth, while single-cell data indicate substantial metabolic variation among endometrioid, serous, and clear-cell malignant states (10, 50, 67). CC exhibits HPV- and hypoxia-associated activation of PI3K-AKT-HIF-1alpha signaling, enhanced glycolysis and lactate export, and glutamine-dependent antioxidant metabolism. Spatial multi-omic studies further show that these programs vary between squamous and adenocarcinoma ecosystems and between tumor cores and invasive regions (47, 68, 69). Therefore, OC is distinguished by ascites/omentum-supported lipid and oxidative flexibility, EC by coupling between host metabolic disease and tumor lipid/glucose programs, and CC by viral-oncogene- and hypoxia-linked glycolytic/glutamine dependence.

### 5.2 Metabolic adaptability and functional impairment of immune cells

Immune-cell metabolic adaptation also differs across the three tumor ecosystems. In OC, ascites-derived and tumor-infiltrating CD8^+^ T cells develop mitochondrial dysfunction, while fatty-acid uptake and lipid peroxidation can promote senescence or exhaustion. Tumor-intrinsic fatty-acid synthesis disrupts infiltrating DCs, and lactate-rich niches favor suppressive macrophage states (42, 53, 70). In EC, cytotoxic T and NK cells encounter glucose competition, hypoxia, and mitochondrial stress, whereas Tregs and TAMs retain function through fatty-acid oxidation, lactate utilization, and lipid scavenging. These pressures may be intensified by obesity, insulin resistance, and hormone-responsive stromal metabolism; the dysfunctional DNAJB1-high NK state and regulatory LAMP3^+^ DC compartment provide single-cell correlates of this metabolic immune failure (11, 15, 50). In CC, highly glycolytic HPV-transformed cells produce lactate and acidification that reduce T- and NK-cell cytokine production, while suppressive myeloid cells consume arginine and generate reactive oxygen/nitrogen species. CD73-adenosine-A2B signaling further induces IL-10 and diminishes effective cytotoxic recognition (71–73). Thus, lipid stress and compartmental nutrient exchange predominate in OC, systemic metabolic disease amplifies immune dysfunction in EC, and lactate/adenosine plus myeloid arginine metabolism are especially prominent in CC.

### 5.3 Metabolite-mediated construction of the immunosuppressive microenvironment

Metabolite-mediated immunosuppression is convergent in mechanism but distinct in relative importance across OC, EC, and CC. In OC, lactate suppresses cytotoxic lymphocytes and promotes macrophage polarization, while IDO1 inhibition can trigger compensatory metabolic adaptation rather than durable immune activation. The CD39-CD73-adenosine pathway is concentrated in activated stroma and exhausted T cells and is associated with chemoresistance and poor outcome (14, 61, 74). In EC, lactate produced by malignant cells can drive M2-like macrophage polarization, and an excess of free fatty acids and lipid-derived stress signals supports tumor growth while impairing effector-cell metabolism (15, 50, 67). Tryptophan-kynurenine and adenosine pathways are plausible additional barriers, but their cellular sources and clinical relevance require stronger EC-specific validation. In CC, glycolysis-derived lactate acts together with CD73-generated adenosine; A2B-receptor signaling increases tumor-cell IL-10, downregulates HLA class I, and protects cells from cytotoxic T lymphocytes (73). ARG1/ROS-producing suppressive myeloid states and IDO1-expressing antigen-presenting cells can further reinforce nutrient deprivation and redox stress (46, 47). Consequently, lactate is a shared suppressor, but OC adds stromal adenosine and adaptive tryptophan metabolism, EC adds systemic lipid/insulin exposure, and CC adds a well-defined adenosine-IL-10-HLA-I escape loop. Therapeutic biomarkers should quantify both the metabolite and its producing/receiving cell populations (**Figure 2**).

**Figure 2:**
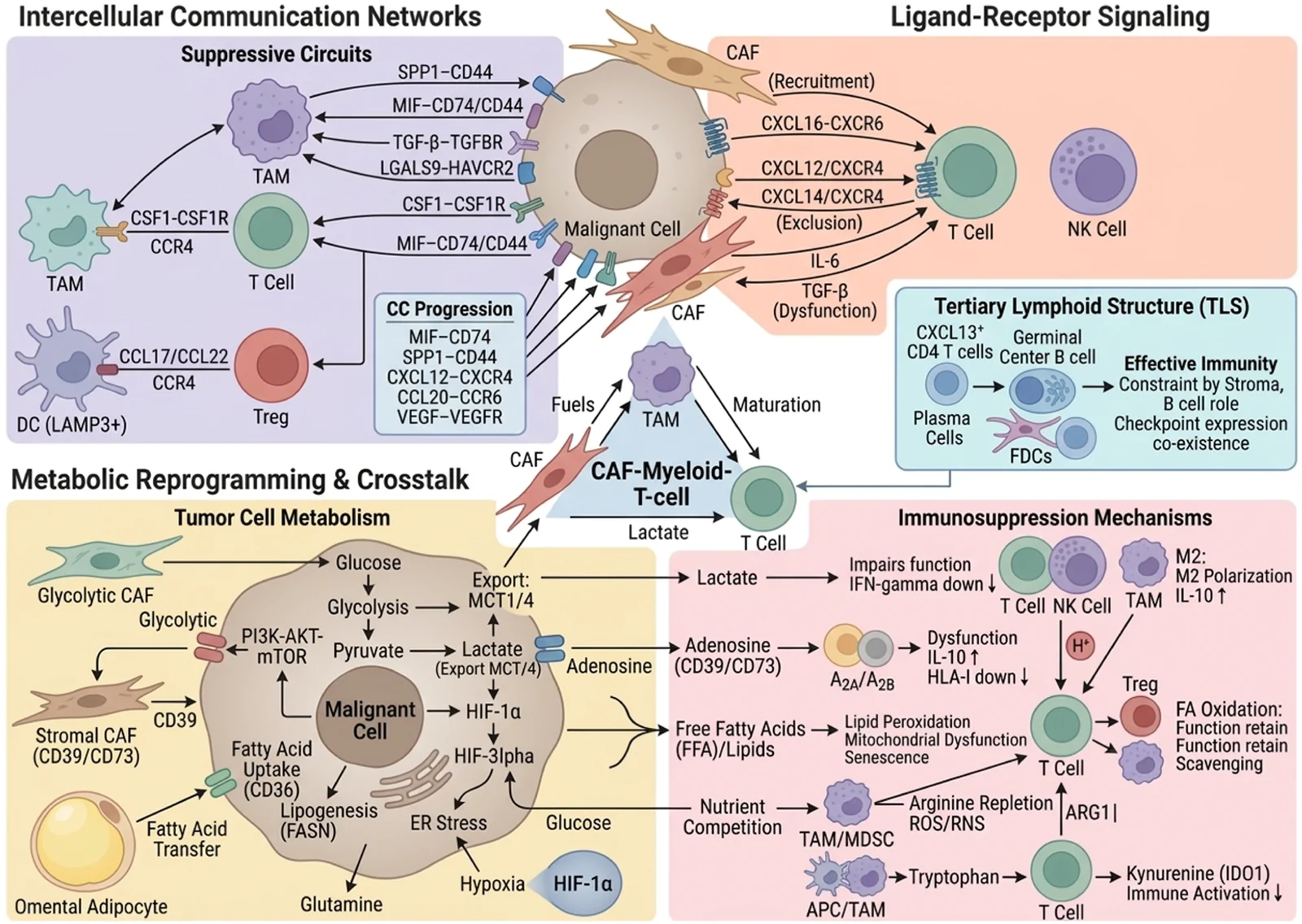
Intercellular communication and metabolic-immune crosstalk in the gynecological malignancy microenvironment.

### 5.4 Novel approaches for single-cell metabolic flux analysis

Conventional metabolic research methods struggle to resolve metabolic heterogeneity at the single-cell level, whereas emerging single-cell metabolic flux analysis approaches now make this feasible. Single-cell RNA sequencing combined with metabolic pathway activity inference algorithms, such as scFEA and Compass, enables estimation of metabolic flux for each cell, predicting metabolic activity without direct metabolite measurements and thereby revealing metabolic preferences of different cell subsets (42). Spatial metabolomics technologies, such as mass spectrometry imaging, integrated with single-cell transcriptomic analysis, enable validation of spatial correspondences between metabolite distribution and metabolic enzyme expression (75, 76). This multimodal integrative analysis strengthens causal inference of metabolic-immune associations, allowing precise localization of metabolite exchange between tumor cells and immune cells in specific regions (77). Furthermore, advances in single-cell proteomics technologies now permit precise quantification of key metabolic enzymes (GLS1, FASN) and transporters (GLUT1, MCT4) at the protein level, bridging the gap between transcriptional profiles and functional proteins and providing more direct information regarding the ultimate effectors of metabolic regulation (78).

## 6 Potential therapeutic targets and clinical translational

Immune-checkpoint therapy has produced unequal clinical benefit across gynecological malignancies, making single-cell-defined resistance mechanisms clinically relevant. In recurrent OC, pembrolizumab monotherapy produced objective response rates of only 7.4-9.9%, consistent with spatial T-cell exclusion, suppressive macrophages/CAFs, and redundant PD-1, TIGIT, LAG3, TIM-3, CD47-SIRPalpha, B7-H3, and adenosine checkpoints (79, 80). Rational development should therefore prioritize biomarker-selected combinations that address both lymphoid exhaustion and myeloid/stromal exclusion. EC provides the clearest genotype-linked sensitivity: dostarlimab achieved substantially higher activity in mismatch-repair-deficient/microsatellite-instability-high than in mismatch-repair-proficient disease, while POLE mutation, tumor mutational burden, TLS, and inflamed T-cell states further refine response prediction (43, 45, 81). For mismatch-repair-proficient EC, LAG3/TIGIT, macrophage, and CAF-directed combinations warrant evaluation. In CC, first-line pembrolizumab plus chemotherapy and later-line cemiplimab have established the value of PD-1 blockade (82, 83). Nevertheless, HPV-driven tumors retain exhausted PD-1^+^TIGIT^+^LAG3^+^ T cells, suppressive myeloid cells, and TGF-beta-rich stroma, supporting combinations directed at coinhibitory receptors, macrophage checkpoints, or CAF-derived signals (20, 47, 64).

In OC, clinical IDO1 inhibition induced compensatory tumor metabolism that constrained antitumor immunity, illustrating why pathway blockade without on-treatment metabolic profiling can fail (74). More defensible combinations include FASN or fatty-acid-uptake inhibition to restore DC/T-cell function, CD39/CD73 or adenosine-receptor blockade to relieve stromal suppression, and lactate-transport inhibition paired with PD-1 blockade or platinum therapy (53, 61). In EC, PI3K-AKT-mTOR signaling, glycolysis, and *de novo* lipogenesis are attractive targets, particularly in metabolically dysregulated or mismatch-repair-proficient tumors. Combining pathway inhibitors with checkpoint blockade may reduce tumor nutrient consumption and improve lymphocyte function, but dosing must avoid simultaneous inhibition of activated immune cells (50, 84). In CC, glutamine metabolism, lactate export, and the CD73-A2B axis provide complementary opportunities: SHP2-dependent glutamine reprogramming and the adenosine-IL-10-HLA-I circuit support testing glutaminase/SHP2, MCT1/4, or adenosine-pathway inhibitors with radiotherapy and PD-1 blockade (69, 73). The preferred combinations therefore differ: lipid/adenosine strategies in OC, insulin-PI3K-mTOR/lipogenic strategies in EC, and lactate/glutamine/adenosine strategies in CC. Prospective trials should incorporate serial metabolomics, pharmacodynamic biopsies, and immune-cell-specific toxicity endpoints rather than relying solely on baseline transcriptional scores.

## 7 Conclusion

Single-cell sequencing technologies have fundamentally transformed our understanding of the gynecological malignancy microenvironment by revealing the profound heterogeneity of tumor-infiltrating immune cells and defining functionally distinct subsets closely linked to prognosis and therapeutic response. These findings challenge conventional classification paradigms, underscoring the necessity of redefining immune subsets based on functional states rather than surface markers alone. Critically, the intricate crosstalk between metabolic reprogramming and immune regulation constitutes a central driver of immune evasion, indicating that immune checkpoint blockade alone is insufficient and must be complemented by metabolic interventions targeting pathways such as glycolysis, glutamine metabolism, or lactate transport. Although single-cell multi-omics analysis have identified numerous potential therapeutic targets and predictive biomarkers, most discoveries remain at the retrospective or preclinical validation stage, lacking confirmation through prospective clinical trials. Moreover, heterogeneity in sample processing, analytical pipelines, and subset nomenclature across studies impedes direct comparability, highlighting an urgent need for standardized protocols and cross-study validation frameworks. Looking forward, the integration of single-cell spatial omics, multi-omics platforms, and functional validation in large clinical cohorts will enable comprehensive spatiotemporal dissection of tumor microenvironment dynamics. These advances are poised to accelerate the translation of molecular insights into precision immunotherapy strategies, ultimately improving outcomes and quality of life for patients with advanced gynecological malignancies.
